# Etiology, Epizootiology and Control of Maedi-Visna in Dairy Sheep: A Review

**DOI:** 10.3390/ani10040616

**Published:** 2020-04-03

**Authors:** Aphrodite I. Kalogianni, Ioannis Bossis, Loukia V. Ekateriniadou, Athanasios I. Gelasakis

**Affiliations:** 1 Laboratory of Anatomy and Physiology of Farm Animals, Department of Animal Science, Agricultural University of Athens (AUA), Iera Odos 75 str., 11855 Athens, Greece; afrokalo@aua.gr (A.I.K.); bossisi@aua.gr (I.B.); 2 Veterinary Research Institute, ELGO-Demeter, Thermi, 57001 Thessaloniki, Greece; ekateriniadou@vri.gr

**Keywords:** maedi-visna virus, ovine progressive pneumonia, small ruminant lentivirus, dairy sheep

## Abstract

**Simple Summary:**

Maedi-visna is a chronic viral disease of sheep with worldwide prevalence. It is caused by a small ruminant lentivirus. Its clinical manifestation includes primarily pneumonia and mastitis, and secondarily, arthritis and encephalitis. It causes substantial economic losses associated with involuntary culling or death of infected animals and reduced productivity, challenging the sustainability of dairy sheep farms. The extensive spread of the disease and the lack of treatment or vaccines render surveillance and prevention strategies indispensable. Currently, the major obstacles in controlling the disease are (i) the absence of an effective protocol for the early and definitive diagnosis of infected animals, utilizing appropriate, universally accepted serological and molecular techniques, (ii) the long interval between infection and seroconversion, and (iii) lack of understanding whether horizontal or vertical transmission are the most important route of infection. Therefore, the most appropriate measures for the control of the disease should include more frequent serological testing with available diagnostics and isolation or culling of seropositive animals, incorporation of artificial suckling, and strengthening of hygiene and biosecurity protocols.

**Abstract:**

Maedi-visna (MV) in sheep is caused by maedi-visna virus (MVV), a small ruminant lentivirus (SRLV) that causes chronic infection and inflammatory lesions in infected animals. Pneumonia and mastitis are its predominant clinical manifestations, and the tissues infected by MVV are mainly the lungs, the mammary gland, the nervous system and the joints. MV has a worldwide distribution with distinct MVV transmission patterns depending on circulating strains and regionally applied control/eradication schemes. Nevertheless, the prevalence rate of MV universally increases. Currently, gaps in understanding the epizootiology of MV, the continuous mutation of existing and the emergence of new small ruminant lentiviruses (SRLVs) strains, lack of an effective detection protocol and the inefficiency of currently applied preventive measures render elimination of MV an unrealistic target. Therefore, modifications on the existing MV surveillance and control schemes on an evidentiary basis are necessary. Updated control schemes require the development of diagnostic protocols for the early and definitive diagnosis of MVV infections. The objectives of this review are to summarize the current knowledge in the epizootiology and control of MV in dairy sheep, to describe the research framework and to cover existing gaps in understanding future challenges regarding MV.

## 1. Introduction

Maedi-visna (MV) (also known as ovine progressive pneumonia, OPP) is an incurable viral disease of sheep with very long incubation period that leads to life-long infection [1]. It is caused by non-oncogenic exogenous retroviruses [2], namely, maedi-visna virus (MVV) and caprine arthritis-encephalitis virus (CAEV), both belonging to a subgroup of viruses known as small ruminant lentiviruses (SRLVs). These viruses are structurally identical, display similar pathogenicity, and have genetic variants/strains that can infect both sheep and goats [3,4]. Species-specific categorization of SRLVs is not always valid since cross-species transmission of certain genotypes is evident with the direction of transmission not being always apparent [4]. Clinical symptoms of SRLVs infections are strain-dependent. Most prevalent are symptoms associated with the respiratory tract, such as dyspnea and abdominal breathing due to chronic interstitial pneumonia. Symptoms associated with encephalitis include instability, paresis, and paralysis. Other clinical manifestations such as mastitis (indurated udder), arthritis (lameness), progressive weakness and, in some cases, death of infected animals may also occur [5]. The primary route of infection for newborn lambs is the consumption of colostrum and milk from infected ewes [3]. Horizontal transmission via respiratory secretions is also significant [4], whereas, vertical transmission (transplacental) [6] and transmission via semen during mating or artificial insemination are also possible [6], but their significance and extent has not been thoroughly investigated.

Recognizing the socio-economic impact of MV, the World Organization for Animal Health (OIE) has included it in the list of notifiable terrestrial and aquatic animal diseases, with significant impact on international trade of animals and their products [7]. In dairy sheep, economic losses associated with the disease are rather extensive. During the last 5 years, the situation seems to have worsened as accumulating evidence suggests an increase in prevalence of the disease especially in the European continent. The scarcity however of epizootiological data regarding inter- and intra- farm transmission of the disease is a major obstacle for the development and implementation of evidence-based disease-control and eradication programs [8]. In particular, in the case of dairy sheep, there is a considerable shortage of certified MVV-free sheep breeding stocks in the international market. This is an obvious drawback in newly established intensive sheep farms, where the interest to buy MVV-free breeding stocks with high productive potential is large. Hence, the need to establish MVV-free dairy sheep farms is now more urgent than ever and forms a major issue for the conservation and evolution of the dairy sheep sector worldwide.

As there is no treatment against MV and all efforts for the development of vaccines did not produce any satisfactory results [6], the control of the disease and the reduction of its prevalence have been achieved to some degree through controlled eradication programs aiming at diagnosis of the infected animals at an early stage. However, these programs are heterogeneous in terms of their planning and effectiveness, which highly depends on the number of participating farms, the regional prevalence of the disease, and the diagnostic methods applied to detect the infected animals [9]. It has now been recognized that early diagnosis of viral infection in MV is more complicated than originally thought and constitutes a major barrier for the effective eradication of the disease [10,11]. Most of the programs used for the control of the disease are based on serological tests to detect antibodies against the virus [12]. This way, only the seropositive animals are diagnosed and removed from the flock. However, many animals infected with the virus remain undiagnosed due to the late (from a few months to three years) seroconversion [13,14]. In the past, several papers have reviewed the status of MV in sheep industry [5,6,15,16,17,18,19,20,21,22]; nevertheless, the objectives of this review paper are to summarize and provide an integrated overview of the current knowledge regarding the epizootiology and control strategies of MV in dairy sheep, to describe the research framework and to cover existing gaps in understanding future challenges regarding MV.

## 2. Current State of the Art in Epizootiology of the Disease

MV was initially described in Iceland in 1939, possibly originating from the importation of live animals from Germany. Although clinical signs were apparent, the situation was likely underestimated or misdiagnosed for many years [5,15]. Eventually, it was transmitted among several countries through the trading of breeding stocks (e.g., Denmark in 1968, Canada in 1970, Hungary in 1972, France in 1976, Norway in 1979, and Finland in 1994) [15].

Nowadays, MV has a worldwide spread, with the exception of Iceland, New Zealand, and Australia which are considered MVV-free regions, but not CAEV-free [23]. Despite the scarcity of MV epizootiological data in the Mediterranean countries with developed dairy sheep sector (Spain, Italy, and Greece), field observations suggest increased seroprevalence [16]. Based on these observations and having recognized the significance of the disease, several European countries (e.g., France, Germany, Netherlands, Italy, Switzerland, and Spain) have applied eradication programs. However, the effectiveness of these programs in controlling the disease has been questionable [6].

The majority of SRLVs epizootiological studies worldwide (Table 1) is limited to primarily small-scale estimation of seroprevalence; nationwide monitoring and active surveillance schemes are scarce as they require a substantial funding which is not available in most cases.

## 3. Etiology

There is a continuous effort for the identification and classification of SRLVs strains due to large genetic diversity of the isolated viruses [17,29]. To date, five strain groups, namely, A, B, C, D, and E with their subtypes have been identified in sheep and goats [1,18]. Group A consists of MVV strains and has 15 subtypes, group B consists of CAEV strains with 3 subtypes, whereas, the other three groups refer to strains which have been isolated from Norway (group C), Spain and Switzerland (group D), and Italy (group E) [18]. Some of the classical strains with known sequences are the K1514 (Iceland) and its neurovirulent clones KV1772-kv72/67 and LV1-1KS1, SA-OMVV (South Africa), EV1 (UK), and P1OLV (Portugal). Many of the fore-mentioned strains have been found to infect both sheep and goats (e.g., all the strains of groups B, C, and D as well as group A subtypes A1–A6, A9, and A11–A13). The subtype A15 has been isolated only from sheep and subtypes A7, A8, A10, A14, E1, and E2 only from goats. The classification of SRLVs, the species infected and the countries first reported are summarized in Table 2.

Recently, phylogenetic analysis revealed a new strain (CRF01_ABSRLV) which was isolated from Canadian goats and possibly originating from the combination of A2 and B1 subtypes [51]. The mechanism for generation of retroviral recombined strains is complicated and not very well understood. However, it could be considered among possible explanations for the emergence of new SRLV strains [51,52].

## 4. Clinical Signs and Gross Pathology

In the majority of cases, virus replication is slow and the number of infected blood cells in the circulation is very low [53]. Therefore, the clinical disease is latent or progressive and in many cases, the clinical signs are not evident or characteristic of the disease at its early stages [3]. Immunosuppression of animals due to aging, co-existing of diseases and environmental stressors accelerates virus replication and clinical evidence of the disease becomes apparent. At the flock-level, serum detection of antibodies, the severity of clinical disease and the number of deaths/culled animals, can be affected by management practices and the co-existence of other diseases [18].

Clinical manifestation of SRLVs infection depends on the virus strain, the host immune response and the host genetic profile regarding resistance or susceptibility to the virus [54,55]. Lesions of SRLVs infection in tissues and organs are caused both by the immune response to the viral antigens and the viral replication itself [3]. The tissues mainly infected by MVV are located at the lungs, the mammary gland, the nervous system, and the joints [3,19]. Pneumonia and mastitis are the predominant clinical manifestations of MVV in sheep [3]. Less frequently, lesions such as lymphoid tissue hyperplasia may be apparent in kidneys, liver, and heart, indicating them as possible target organs [56,57]. Multiple-organ infection may be observed in the progression of the disease, but the severity of lesions varies among the affected organs [19].

Respiratory clinical signs include dyspnea and increased respiratory rate, caused by the characteristic lymphocytic interstitial pneumonia; at necropsy, the lungs appear discolored, enlarged, and diffusely firm with grey spots on the pleural surface and the mediastinal lymph nodes are often enlarged [19,23].

Symptoms from the nervous system include ataxia, paresis, weakness in hind limbs, incoordination or, in heavier cases, total paralysis, due to meningoencephalitis, astrocytosis, microgliosis, and focal secondary demyelination in the brain and the spinal cord [3,19].

In mammary gland, MVV can cause an indurative non-suppurative interstitial mastitis [3,19]. The udder is hard but not painful, with decreased milk production mainly noticed the first days postpartum, a situation usually described as “hard udder syndrome”. The lymphocytic inflammatory pattern, caused by the replication of virus in macrophages and mammary gland epithelial cells, provokes the destruction of the acinar structure and the reduction of milk production [19]. 

Arthritis can also be the outcome of MVV infection, although it is less common in sheep [3,16,18,19]. The affected joints are usually the carpal and tarsal, but metatarsal, metacarpal and vertebral joints can also be affected [16,18,19]. Infiltration of the synovial membrane by mononuclear cells is followed by villous hypertrophy, angiogenesis, and finally fibrosis, mineralization, and necrosis of synovium and joint capsule [3]. In advanced arthritis cases, the cartilage is destructed and the articular capsule is fibrotic [18,19]. In the majority of cases, arthritis is progressive causing lameness and involuntary culling of the animal, whereas less often it may regress [3].

## 5. Transmission

The mechanisms and the significance of horizontal and vertical transmission of the MVV have not been fully clarified yet [3]. The major routes of transmission have been described; however, their significance and extent remain unclear. This information is critical for the efficient designation of the eradication protocols, especially in intensively reared dairy sheep. 

The vertical transmission of MVV refers to the transmission of the virus from the ewe to the lamb during pregnancy (transplacental), at lambing or during suckling [6]. Opinions regarding transplacental transmission are controversial [15,58]. Seropositivity of 4- to 9-months old lambs derived by caesarean section, isolated after birth and not allowed to suckle seropositive dams support the transplacental transmission [59]. However, the possibility of horizontal transmission post-lambing, complicates the assessment of the significance of either transplacental or horizontal transmission [6,15]. Vertical transmission at lambing refers to the transmission of virus while the lamb passes through the ewe’s genital tract and it is exposed to maternal body fluids and blood. The exact significance of this route of transmission remains unknown [6,58].

The most significant route of vertical transmission is considered to be the lactogenic, through the ingestion of colostrum and milk from infected dams [6]. MVV shows tropism to the epithelial cells of the mammary gland and the resident macrophages, where it can replicate [60]. It has been found that isolated lambs fed colostrum or milk from infected ewes seroconverted a few months later and some of them were diagnosed with clinical disease later in their adult life [15]. There is evidence that the lactogenic transmission is more significant in small ruminants than in primates due to the higher permeability of the digestive tract of small ruminants in the first 24 h post-lambing [61,62], allowing virions and infected cells to be absorbed by the lamb’s intestine [62]. However, not all the subgroup variants of MVV are efficiently transmitted via the lactogenic route as the envelope varies among the different subgroups of the MVV determining some of its physicochemical properties and facilitating or not the lactogenic transmission [62]. 

Horizontal transmission of MVV includes the environmental, mechanical and iatrogenic routes and mainly refers to the transmission through respiratory secretions [63]. Lungs are the main target organ of MVV in the respiratory tract. In lungs, the virus infects monocytes, macrophages, and dendritic cells, and it can be horizontally transmitted via respiratory secretions containing these cells [3,15]. In severe cases, MVV causes the characteristic lesions of interstitial pneumonia. In general, the lower respiratory tract, constitutes the main route of infection [15,64]. This route of transmission is of major importance in intensive and permanently housed sheep in sheds with inadequate ventilation and high stocking density [19]. In general, many researchers support that the airborne transmission can be an equally significant route of transmission as the vertical transmission. For this reason, the segregation of newborn lambs or non-infected animals from the infected ones is of major importance for the control of MVV transmission [3,15,65]. The significance of transmission through contaminated barns, sheds, feeding and water equipment and pastures or reusable veterinary equipment has not been fully clarified [6,15]. The presence of the virus in the water and air from pens with infected animals [63] indicates that waterborne and airborne transmission in the farms cannot be disregarded. Infection of dairy sheep via the teat canal during milking has also been reported [15].

Sexual transmission of the MVV is theoretically possible but not yet confirmed. However, there is evidence of virus proliferation in the genitals of infected rams and the virus has been found in the semen of rams with leucocytospermia and rams positive for *Brucella ovis* [3,66]. In another study using real-time PCR, proviral DNA of SRLV was found in semen (intermittent shedding) and the genital tract of rams suggesting possible sexual transmission [67]. 

## 6. Risk Factors

There are several risk factors that influence transmission of MVV between and within flocks. These factors determine the likelihood of infection, the prevalence, the incidence rate and other epizootiological characteristics of the disease. Identification and mitigation of risk factors at the farm level is therefore crucial when establishing a MVV control/eradication program. Flock size/stocking density, intensity of the farming system [9,10,11,12,26,27], and age distribution [9,10,11] affect the likelihood of seropositivity at flock level, indicating the significant role of horizontal transmission in the epizootiology of MV [9]. For example, lower prevalence in extensively reared sheep can be attributed to the reduced stocking rates and limited direct contact between animals [68,69], conditions that reduce the exposure to MVV and the possibility of airborne transmission through respiratory droplets during exhalation, sneezing, and coughing [3,6]. In flocks where MV co-exists with pulmonary adenomatosis, the transmission is favored by the increased quantities of respiratory secretions produced by infected sheep [9,15]. In these cases, late removal of clinical cases of MV and non-isolation of seropositive animals are significant risk factors for the transmission and increased seroprevalence of the disease.

Inappropriate cleaning and disinfection of milking equipment [15,19], reuse of infected needles and surgical equipment, inadequate hygiene conditions inside the barn and grazing at common pasturelands are also potential risk factors for the horizontal transmission of MVV. 

Importation of breeding stocks from flocks of unknown MVV-status is associated with increased seroprevalence of MV [10,27]. The remarkable absence of certified MVV-free flocks to produce breeding stocks and the use of seropositive rams for mating or artificial insemination are the main causes. Surprisingly, despite the lactogenic transmission of the virus through colostrum/milk during suckling, a reduced seroprevalence in the replacement stocks has been observed in flocks with increased suckling period [9]. This is possibly the result of a confounding effect of farming system; increased weaning age is mainly observed in semi-extensive and extensive systems, where horizontal transmission is limited. On the other hand, early weaning is mainly practiced in intensive systems where virus transmission is facilitated mostly due to the permanent housing, the increased stocking density and the inappropriate ventilation [9,10,70]. Nevertheless, the use of colostrum/milk from seropositive dams and natural suckling of newborn animals constitute major risk factors. Also, in mixed-species flocks (sheep and goats) the seroprevalence has been found to be higher, possibly due to cross-species transmission of several SRLVs strains [10,70]. 

There is evidence of genetic resistance/susceptibility against SRLVs [71]. Different alleles of the cellular *TMEM154* (*Transmembrane protein 154*) gene have been found to be associated with the occurrence of MV. Haplotypes carrying nucleotide sequences that code for the amino acid glutamate at position 35 are associated with increased susceptibility to MV, whereas haplotypes carrying nucleotide sequences that code for lysine at the same position are associated with resistance to MV [72,73,74]. Also, the haplotype responsible for the susceptibility seems to be dominant against the “resistant” haplotype [74]. Although there is indication for association between *TMEM154* mutations and control of MVV infection, there is no proven association for all the haplotypes [73]. Other genes associated with virus susceptibility are the *DPPA2* (*Developmental Pluripotency Associated 2*)/*DPPA4* (*Developmental Pluripotency Associated 4*), *SYTL3* (*Synaptotagmin-Like 3*), *CCR5 (Chemokine receptor 5)*, MHC (Major Histocompatability Complex), *TLR7, TLR8, TLR9* (*Toll-like receptors*) genes, and APOBEC3 (Apolipoprotein B mRNA-editing enzyme) proteins [75,76,77], whereas the zinc finger cluster, *C19orf42* (*Chromosome 19 Open Reading Frame 19*)/*TMEM38A* (*Transmembrane Protein 38A*) and *DLGAP1* (*Discs Large (Drosophila) Homolog-Associated Protein 1*) genes may be used in genetic selection programs to facilitate the control of the disease [78]. Τhe tripartite motif-containing 5 (TRIM5) protein has been studied and has been proved to contribute to the restriction of MVV [79].

## 7. Diagnosis

Early and efficient diagnosis of MV is a critical parameter for the control and eradication of the disease. The diagnosis of SRLVs infections is based on the detection of antibodies against the virus proteins or the viral genome. Current control and eradication programs are based on serological tests (mainly enzyme-linked immunosorbent assays (ELISAs)) to detect antibodies against the virus [13]. Therefore, only the seropositive animals are considered infected and subsequently removed from the flock. This is a major drawback for the eradication of the disease as the immune response to the disease (seroconversion) requires a long period of time and thereby, many of the infected animals remain undiagnosed carriers of the virus [8,14]. The available assays for the detection of antibodies are the agar gel immunodiffusion (AGID) test, radioimmunoprecipitation assay (RIPA), Western blotting (WB) and ELISA, whereas, polymerase chain reaction (PCR) is used for the detection of proviral DNA as described below.

### 7.1. AGID Test

AGID test is commonly used as a diagnostic tool in MV control programs due to its simplicity. It is a highly specific diagnostic method but less sensitive than ELISA [6,19,20,21]. For this reason, it is supplementary used for the confirmation of the ELISA test [20].

### 7.2. RIPA

RIPA is as old as WB and both of them are considered as the reference standards. They have similar sensitivity and are mainly used as confirmatory assays [21,22]. RIPA is not frequently used due to its high cost and its difficult application.

### 7.3. WB

WB is a confirmatory laboratory test which has been used to detect antibodies in serum that recognize viral proteins. In general, WB is more sensitive than the ELISAs but more cumbersome and with lower throughput [6,21]. Cross-reactivity with non-specific cellular proteins is also a problem.

### 7.4. ELISA

ELISA is the most commonly used test in population screening and for the surveillance of SRLVs. It detects antiviral antibodies with satisfactory sensitivity and specificity, indicating the occurrence of infection and seroconversion at some point [6,20]. Seropositivity is not necessarily followed by clinical disease and a seronegative animal cannot be safely considered to be free of infection. In many cases, seroconversion requires several months even in early-infected animals (e.g., lambs infected via colostrum consumption) [17]. Except for this hurdle, the antigenic heterogeneity of SRLVs strains (especially among different subtypes, like the CAEV-like and the MVV-like) may limit the diagnostic performance of currently available ELISA [80]. Antibody titers present remarkable variations during an animal’s life and in some cases, they are undetectable using an ELISA, which renders the test unreliable for a definitive diagnosis [17,21,22,81].

### 7.5. PCR

PCR can directly detect proviral DNA in fluids and tissues across the animal body (lungs, milk, peripheral blood, mammary gland, synovial membranes etc.). The most reliable cells for the detection of virus are the peripheral blood mononuclear cells [20,21]. The most significant advantage of PCR is its ability to detect infection before seroconversion. However, PCR is not a reference method and it is suggested to be combined with serological testing to overcome the problem of selective specificity associated with the lack of reliable universal primers [6,20,21]. PCR nested methods and Real-Time-PCR (RT-PCR) increase the sensitivity and specificity of the method, however, their use is less frequent [20,21,82].

## 8. Treatment–Vaccination

There is neither a treatment nor an effective vaccine against MV. In the past, there have been attempts for the development of attenuated and subunit vaccines but none of them proved to be effective in preventing viral infections [83,84,85,86,87,88,89]. The major obstacles for the development of an effective MV vaccine include the necessity for the induction of high antibody titers against the virus, the wide genetic variation of viral strains and its continuous mutations, the increased post-infection immunological reaction, the post-vaccination challenge on the immune system and the evidence that the production of SRLV-specific neutralizing antibodies, following vaccination with whole virus-, protein-, and live attenuated-vaccines, is not always protective and in some cases may favor persistent infection [3,54]. The last obstacle is correlated with the fact that the relationship of antibody production with the protection against the SRLVs infection is doubtful and the cellular immune response seems to be more critical [3,54]. Current research efforts for the development of an effective vaccine include pseudoviruses/viral particles, recombinant viruses carrying genes from MVV, and naked plasmids carrying MVV genes plus factors enhancing innate immune responses. However, the effectiveness of these alternative strategies has not been sufficiently validated and thus are considered inappropriate for commercial use [20,54].

## 9. Preventive and Eradication Measures

The preventive measures and management interventions that could aid in controlling or eradicating MV should be decided on case by case and include the following:

1. Annual, biannual, or more frequent blood sampling from the breeding stocks and serological and molecular testing for the diagnosis of the infected animals.

2. Post-lambing management primarily based on the application of artificial suckling and the use of colostrum and milk substitutes or pasteurized colostrum/milk. The management of colostrum involves the administration of bovine colostrum, commercial sheep colostrum or colostrum only from uninfected ewes or heat-treated colostrum (56 °C for 60 min) [6,20,90]. Artificial suckling should take place in an area isolated from adult animals, which has to be regularly cleaned and disinfected. 

3. Immediate removal of animals with apparent clinical signs and positive laboratory diagnosis. The selective culling of these animals and their replacements with seronegative animals, or the grouping of animals according to their seroconversion status can be applied in areas with moderate seroprevalence [6,20,91]. The later strategy requires the spatial and managerial separation of seropositive and seronegative groups within the farm [20,91]. In flocks with high seroprevalence, the most efficient practice is the annual culling of the oldest and less productive seropositive animals and their exclusive replacement with seronegative breeding stocks. The selective culling of seropositive animals would not facilitate the rapid control of the disease, but can contribute to the reduction of seroprevalence and infection rate at flock-level, enhancing the potential of a more drastic elimination program in future time (i.e., culling of the remaining seropositive animals) [9,20]. 

4. Keeping the replacement animals, post-weaning, in separate housing facilities to avoid horizontal transmission of MVV through the contact with adult animals of the remaining flock.

5. Purchased animals should be from certified MVV-free farms. Imported animals need to remain on quarantine until the MV-status is determined using the most appropriate assays.

6. Regular cleaning and disinfection of facilities and equipment with appropriate disinfectants. The cleaning and disinfection schedule must include the barn (floor, walls, bedding), the milking machine, the feeders and the waterers. 

7. Reduction of stocking density (sufficient area and volume) and adequate ventilation. 

8. Implementation of general good hygiene practices. Use of disposable needles or sterilization of metal needles before their reuse is necessary. Similarly, the medical equipment should be sterilized after its use. 

9. MV seronegative milking ewes should be grouped separately and machine-milked before the seropositive ones. 

10. Grazing in communal pastures and sharing of infrastructures and equipment should be avoided when the MVV-status of the flocks is unknown.

11. Rams used either for mating or for semen collection need to be MVV-free [92]. Currently, attempts are being made to produce SRLVs-free breeding stocks via reproductive biotechnologies like artificial insemination and embryo transfer even from infected males and females, respectively. In the case of embryo transfer, this may be possible via the removal of cumulus oophorus cells [92]. 

12. Breeding for resistance could also be considered, but universally accepted resistant genotypes are yet to be developed [18]. 

The characterization of a flock as MVV-free demands two to five successive negative tests every 6 months, yearly or every 2 years (depending on the country). The trade of live animals is allowed when the animals have no clinical signs of MV, the adult animals are seronegative and MV has neither been clinically nor serologically diagnosed in the sheep flocks of origin during the last three years. Also, artificial insemination is allowed only using semen from seronegative rams. According to OIE, a country is considered MVV-free when <1.0% of herds are infected with 99.0% probability.

After the first implemented eradication program in Iceland, a lot of countries applied their own eradication programs (almost all the European countries and Canada) with variable results. The major obstacles for the successful implementation of eradication programs are (i) the possible refusal of farmers’ participation [6,9], (ii) the breed variability, which, as previously mentioned associates with susceptibility and resistance against the disease, (iii) the genetic variability of the viral strains and the different epizootiological characteristics of the disease (virulence, transmission, seroconversion, seroprevalence at flock level, etc.) and (iv) the heterogeneous farming and herd health management systems. For this reason, the eradication program needs to be adjusted and optimized according to the fore-mentioned factors. An indicating classification of the flocks according to the observed seroprevalence could be: flocks with high (>70.0%), medium (40.0%–69.0%), low (10.0%–39.0%), very low (1.0%–9.0%) seroprevalence and the MVV-free flocks (<1.0%) [6,20]. 

The eradication measures when considered in a country-wide scale should include both species of small ruminants due to the fact that the cross transmission has been proven and is a significant risk factor in the spread of the virus [89]. Moreover, the existence of reference laboratories for the control of MV is of major importance [20]. These laboratories will be responsible for the surveillance of MV at national level and will coordinate all the efforts for the elimination of the disease [6]. 

Total replacement of seropositive flocks with breeding stocks from MVV-free flocks could be a sustainable option only in areas where the seroprevalence is very low and MVV-free flocks are available. Otherwise, there is a serious threat for significant monetary losses and restriction of genetic resources which may undermine the sustainability of the farms, particularly in areas with developed dairy sheep farming industry [20].

## 10. Conclusions and Future Challenges in Controlling MV

Epizootiological investigations regarding SRLVs infections are essential for the territorial mapping of virus distribution. The scarcity of detailed epizootiological data, in combination with the continuous mutations of SRLVs strains, render the creation of a universal, reliable and valid diagnostic protocol, and consequently the control of SRLVs, a rather complicated task. 

For the early and definitive diagnosis of SRLVs infections, development of appropriate combinations of serological and molecular tests (ELISAs and PCR) on an evidential basis is suggested and forms a challenging research field in the future. 

Determining the epizootiological traits of SRLVs infections and particularly the significance of the different routes of viral transmission will facilitate the decision-making process towards the determination of the most effective measures for the creation of MVV-free flocks. The global spread of SRLVs highlights the underestimation of the problem by the farmers and the insufficient implementation of the suggested preventive measures, which mainly focus on the lactogenic and airborne transmission of the disease, but may also be indicative of the failure in controlling the disease using these measures. Therefore, the significance of every other possible route of transmission (transplacental, sexual, and iatrogenic) needs to be carefully assessed and revised, and relevant preventive measures need to be integrated in the MV control protocols. 

Exploitation of active surveillance programs and quantification of the consequences of SRLVs’ infection on production [93], health and welfare traits are critical endeavors to determine the overall impact of the disease on the sustainability and resilience of the farms. For this reason, large-scale longitudinal and cohort studies are necessary to collect data for the determination of the most appropriate prediction models estimating the cost of the disease (production losses, involuntary culling/increased replacement rate, predisposition to other diseases and control measures), which remains unknown.

Breeding for resistance against SRLVs is an emerging research field. Specific genes have already been found to be associated with resistance or susceptibility to the disease and the discovery of additional genomic regions may increase the options for its control through marker-assisted selection (MAS) [76].

Despite the significant advances accomplished in SRLVs immunization studies, there are still important challenges to address, as vaccination against SRLVs may contribute to either controlling or enhancing MV [54]. Therefore, research efforts on the field of immunization have to be focused on the development of safe and effective vaccines, with the potential of universal application and mass production.

## Figures and Tables

**Table 1:** Epizootiological studies regarding small ruminant lentiviruses (SRLVs).

Region	Species	Seropositivity at Animal Level (%)	Seroprevalence at Flock Level (%)	Number of Flocks	Number of Animals	Reference
Spain (Aragon)	s	52.8	100.0	554	274,048	[9]
Spain (Northwestern)	s	25.0	53.0	78	15,155	[10]
Italy (Southern)	g	18.64	51.69	1060	4800	[24]
Greece	s	41.96	Ν/A	6	143	[25]
Belgium	s g	9.0 6.0	17.0 13.0	87 76	555 401	[11]
Switzerland	s	9.0	N/A	241	5084	[12]
Poland (Central Eastern)	s	10.2	N/A	98	2925	[26]
Turkey (Central Anatolia)	s g	9.3 7.5	N/A	N/A	279 146	[14]
Turkey (Instanbul)	s	15.3	N/A	4	542	[14]
Germany (Mecklenburg Western-Pomerania)	s	28.8	51.2	41	2229	[8]
Canada (Manitoba)	s	2.5	25.1	77	2207	[27]
China (Yunnan, Guizhou, Gansu, Ningxia, Shandong, Sichuan, Hunan, Guangdong, Chongqing, Guangxi, Jilin, Anhui)	s	4.6–50.0	N/A	24	672	[28]

s: sheep, g: goats, N/A: relative data is not available.

**Table 2:** Classification of SRLVs strains.

Group	Subtype	Strain	Origin Country	Species	Reference
A	A1	P1OLV	Portugal	s/g	[30]
		KV1772	Iceland	s/g	[31]
		LV1-1, KV1514	Iceland	s/g	[32,33]
		EV1	England	s/g	[34]
		SA-OMVV	South Africa	s/g	[35]
	A2	85/34	USA	s	[36]
	A3	697	Spain	s/g	[37]
	A4	SRLV-A4	Switzerland	s/g	[29]
	A5, A6, A9, A11-A13			s/g	[17,18]
	A7, A8, A10, A14			g	[17,18]
	A15			S	[17,18]
	A16 and A17		Poland	g	[38]
B	B1	FESC-752	Mexico	s/g	[39]
		CAEV-CO	USA	s/g	[40,41]
		GANSU	China	s/g	[42]
		SHANXI	China	s/g	[43]
	B2	S-496	Spain	s/g	[44]
	B3	Volttera and Fonni	Italy	s/g	[45]
	B4		Canada	g	[46]
C		1GA	Norway	s/g	[47,48]
D			Spain, Switzerland	s/g	[17,18]
E	E1	Rocaverano	Italy	g	[49,50]
	E2	Seui	Italy	g	[49,50]

s: sheep, g: goats.
